# Evidence for Positive Selection in the C-terminal Domain of the Cholesterol Metabolism Gene *PCSK9* Based on Phylogenetic Analysis in 14 Primate Species

**DOI:** 10.1371/journal.pone.0001098

**Published:** 2007-10-31

**Authors:** Keyue Ding, Samantha J. McDonough, Iftikhar J. Kullo

**Affiliations:** Division of Cardiovascular Diseases, Mayo Clinic and Foundation, Rochester, Minnesota, United States of America; Indiana University, United States of America

## Abstract

**Background:**

Cholesterol homeostasis is maintained through finely tuned mechanisms regulating intestinal absorption, hepatic biosynthesis and secretion as well as plasma clearance. Proprotein convertase subtilisin/kexin type 9 (PCSK9) is a secreted enzyme of the serine protease family that reduces cellular uptake of plasma low-density lipoprotein (LDL) cholesterol by promoting LDL receptor (LDL-R) degradation. Species-specific positive selection has been noted in the *LDLR* promoter, leading to differential expression of *LDLR* among primates. Whether *PCSK9* experienced significant selective pressure to maintain a functional relationship with its target protein, LDL-R, is unknown.

**Methodology/Principal Findings:**

We compiled the sequences of the coding regions of *PCSK9* from 14 primate species in the clade of Hominoids, Old World monkeys and New World monkeys. To detect selective pressure at the protein level, the ratios of nonsynonymous/synonymous substitution rate (*d*_N_/*d*_S_) under different evolutionary models were calculated across the phylogeny of *PCSK9*. Maximum likelihood analyses of *d*_N_/*d*_S_ ratios for the aligned coding region sequences among 14 primate species indicated that *PCSK9* was subject to a strong functional constraint (i.e., purifying selection). However, positive selection was noted in the functional carboxyl-terminal (C-terminal) domain in many branches across the phylogeny, especially in the lineage leading to the orangutan. Furthermore, at least five positively selected amino acids were detected in this lineage using the branch-site model A. In a sliding-window analysis, several *d*_N_/*d*_S_ peaks in the C-terminal domain in both the human and the orangutan branches were noted.

**Conclusions:**

These results suggest that among primates, differential selective pressure has shaped evolutionary patterns in the functional domains of *PCSK9*, an important regulator of cholesterol homeostasis.

## Introduction

**Figure 1:**
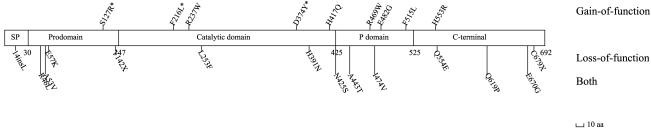
Distribution of non-synonymous variations along *PCSK9*. There are five functional domains in the *PCSK9* protein [4]–[6]: 1) a signal peptide (SP) (1∼30 aa), 2) a prodomain (31∼147 aa), 3) a catalytic domain (148∼425 aa), 4) a putative P domain (426∼525 aa), and 5) a C-terminal domain (526∼691 aa). Gain-of-function mutations are only identified in families with hypercholesterolemia [10] or subjects with high LDL cholesterol levels [17], and loss-of-function mutations in subjects with low levels of LDL cholesterol [15]–[19]. Some non-synonymous mutations have been identified in subjects with either high or low plasma LDL cholesterol [4], [17], and are labeled ‘both’ in the figure. Rare mutations found in families with autosomal dominant hypercholesterolemia are labeled with an asterisk. The gain-of-function mutations are: S127R, F216L, R237W, D374Y, H417Q, R469W, E482G, F515L and H553R. The loss-of-function mutations are: 14insL, E57K, Y142X, L253F, H391N, Q554E, and C679X. Mutations associated with either high- or low- plasma levels of LDL cholesterol subjects are: R46L, A53V, N425S, A443T, I474V, Q619P, and E670G.

The low-density lipoprotein (LDL) receptor gene (*LDLR*) plays a key role in cholesterol homeostasis by receptor-mediated endocytosis of LDL cholesterol. Proprotein convertase subtilisin/kexin type 9 (*PCSK9*, MIM 607786) – a secreted enzyme of the serine protease family – is a newly discovered regulator of *LDLR* [1]–[3]. The human *PCSK9* locus spanning 25 kb and containing 12 exons, resides on chromosome 1p32. The *PCSK9* protein contains five functional domains, a signal-peptide and prodomain at its N-terminus, followed by a catalytic domain, a putative domain and a cysteine-rich carboxy-terminal domain [4]–[6] (Figure 1). *PCSK9* induces LDL-R breakdown [7], internalization and recycling [8], [9] and thereby reduces LDL clearance, and increases plasma levels of LDL cholesterol. Overexpression of wild-type *Pcsk9* gene in mice results in reduced number of LDL-R and hypercholesterolemia [4], [8], [9].

Mutations in *PCSK9* can cause severe autosomal dominant hypercholesterolemia [4], [8]–[14] (i.e., ‘gain-of-function’ mutations), and also low circulating levels of LDL cholesterol [15]–[19] (i.e., ‘loss-of-function’ mutations). Kotowski et al. [17] described a spectrum of nonsense/missense mutations in *PCSK9* that were associated with low or elevated LDL cholesterol levels, in both black and white subjects. Relatively common sequence variants in *PCSK9* also contribute significantly to inter-individual variation in plasma levels of LDL cholesterol in the general population [17]. Cohen et al. [20] showed that two nonsense mutations (Y142X and C679X) in blacks, and one missense mutation (R46L) in whites, were associated with reduced plasma levels of LDL cholesterol and lower incidence of coronary heart disease events. We have summarized these non-synonymous variations in *PCSK9* in Figure 1.

Extant primates show a wide range of phenotypic adaptations to diverse environmental conditions, including substrate and diet [21]. Significant differences in lipid profiles occur among primates; for example, New World and Old World monkeys have significantly lower serum total cholesterol, triglycerides, and LDL cholesterol levels than Hominoids [22]. Among Hominoids, gorillas have the highest circulating total cholesterol, triglycerides, and high-density lipoprotein (HDL) cholesterol levels [22]. Caceres et al. [23] found that several genes related to lipid metabolism were differentially expressed in humans and non-human primates. *LDLR* has also been shown to be differentially expressed among mammals [24].

Activation of the sterol regulatory element binding protein-2 (SREBP-2), a key transcription factor of *LDLR*, not only leads to increased expression of *LDLR*, but also of *PCSK9* [25], and the mRNA expression of *LDLR* and *PCSK9* are coordinately up-regulated in absence of sterols [26]. The dual regulation of *LDLR* [26] suggests that *PCSK9* might be involved in a co-evolutionary network of LDL cholesterol metabolism, i.e., variation at one gene evolving with variation at the other. *PCSK9* is only found in vertebrates, suggesting it is the product of recent evolution [27]. However, it is unclear whether natural selection has driven the evolution of *PCSK9* in vertebrates, especially in primates.

The ratio of nonsynonymous/synonymous substitution rate (i.e., ω = *d*_N_/*d*_S_) provides a sensitive measure to detect selective pressure at the protein level [28]. A significantly higher non-synonymous substitution rate than synonymous substitution rate (i.e., *d*_N_/*d*_S_>1) is evidence for adaptive evolution at the molecular level, whereas *d*_N_/*d*_S_<1 suggests purifying selection (i.e., selective constraint) [29]. This criterion has been used to identify several cases of positive selection, such as the primate stomach lysozyme (*LYZ*) [30], [31], and *BRCA1* in humans and chimpanzees [32] (for a list of genes under positive selection in the human lineage, see review by Sabeti et al. [33]).

In the present study, we used a phylogeny-based maximum likelihood method to analyze nonsynonymous and synonymous substitution rate of *PCSK9* sequences across a range of primates, including Hominoids, Old World monkeys, and New World monkeys. We aimed to address three questions: 1) whether the ratio of nonsynonymous and synonymous evolutionary rate of *PCSK9* varied significantly among various primate lineages, 2) whether there is an episodic adaptive evolution of *PCSK9* in primates, and 3) which, of any, amino acids of *PCSK9* were under positive selection.

## Results

### Comparative analysis of coding regions of PCSK9

**Figure 2:**
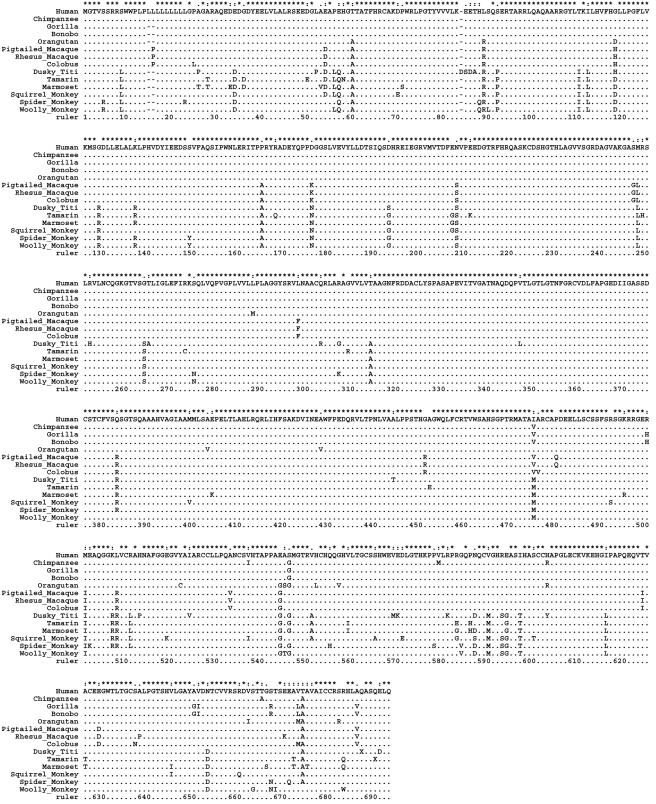
Protein sequence alignment of *PCSK9* in 14 primates. “.” Indicates identity to the first sequence (i.e., human) in each alignment. “-” indicates an alignment gap, and “X” indicates a stop codon. The coordinates after 84 should be minus one to be consistent with that in Human reference sequence (NP_777596), since an insertion at position 84 was present in the dusky titi. The signal peptide (SP) domain (1–90) shows the evolution of Leucine (Leu) repeats (15–23) in *PCSK9*, and the C-terminal domain shows the premature stop codon (X) in the tamarin (686), and dusky titi (689).

We characterized the coding regions of *PCSK9* in human and non-human primates (Table 1). There was considerable evidence for interspecies genomic alterations within *PCSK9* (Figure 2). First, in the signal peptide domain, a variable number of CTG codons [encoding Leucine (Leu, L)] were identified (Figure 2). A species-specific nine Leu repeat (L9) was noted only in humans and chimpanzee; whereas in other species, the number of Leu repeats varied from L6 to L8. In the CTG repeat region, interspecies nucleotide differences were synonymous except nonsynonymous changes in the Old World monkeys (CTG→CCA, 46→48) and in spider monkey (CTG→CGG, 67→69). Second, in the C-terminal domain, species-specific loss-of-function mutation was seen in the clade of New World monkeys (via a premature stop codon in tamarin and dusky titi). We speculate that this nonsense mutation leads to a loss-of-function since the adjacent C679X mutation in humans is a loss-of-function mutation.

**Table 1:** A listing of the primates in this study.

Species	Scientific name	Lineage	Accession number
Human	*Homo sapiens*	Hominoid	EF692496
Chimpanzee	*Pan troglodytes*	Hominoid	EF692497
Bonobo	*Pan paniscus*	Hominoid	EF692498
Gorilla	*Gorilla gorilla*	Hominoid	EF692499
Orangutan	*Pongo pygmaeus*	Hominoid	EF692500
Pigtailed macaque	*Macaca nemestrina*	Old World Monkey	EF692501
Rhesus macaque	*Macaca mulatta*	Old World Monkey	EF692502
Colobus	*Colobus guereza*	Old World Monkey	EF692503
Dusky titi	*Callicebus moloch*	New World Monkey	EF692504
Tamarin	*Saguinus labiatus*	New World Monkey	EF692505
Marmoset	*Callithrix jacchus*	New World Monkey	EF692506
Squirrel monkey	*Saimiri boliviensis*	New World Monkey	EF692507
Spider monkey	*Ateles geoffroyi*	New World Monkey	EF692508
Woolly monkey	*Lagothrix lagotricha*	New World Monkey	EF692509

### Variable d_N_/d_S_ ratios for the C-terminal domain of PCSK9 among primate lineages

A neighbor-joining (nj) phylogenetic tree of *PCSK9* from 14 primate species was reconstructed from the coding sequence alignment, and the maximum likelihood estimate of the tree topology was acquired using the ‘hyphy’ package [34]. We used this gene tree in subsequent analyses to detect whether non-neutral evolution might have operated on *PCSK9*.

Under a one-ratio model, which assumes the same *d*_N_/*d*_S_ for the entire tree, the cumulative *d*_N_/*d*_S_ for the coding regions of *PCSK9* was 0.186 (the log likelihood value *l*_0_ = −5109.09). We tested variable *d*_N_/*d*_S_ ratios for *PCSK9* among lineages, using the free-ratio model, which assumes a different *d*_N_/*d*_S_ ratio for each branch in the phylogeny (Figure S1). The free-ratio model led to a log likelihood *l*_1_ = −5097.59. The free-ratio model was not found to be significantly better than the one-ratio model (the statistic 2Δ*l* = 2(*l*_1_−*l*_0_), *P* = 0.480, degrees of freedom (df) = 24).

**Figure 3:**
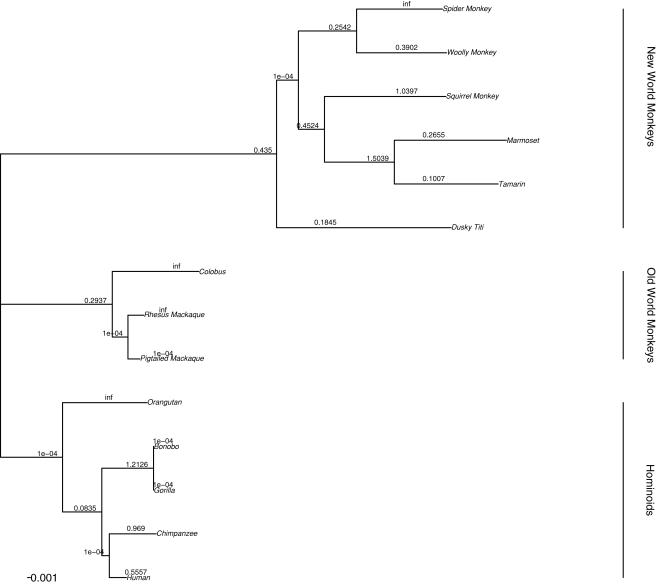
Ratios of *d*_N_/*d*_S_ estimated for the C-terminal domain of *PCSK9* in indicated branches of the primate phylogeny. Values of *d*_N_/*d*_S_ along each branch were calculated by using the free-ratio model using the CODEML program in ‘PAML’ [72]. Branch lengths were estimated by maximum likelihood under this model. A *d*_N_/*d*_S_ value of >1 suggests that positive selection has acted along that lineage. ‘Inf’ indicates cases where *d*_S_ = 0. The phylogenetic tree was deduced from the entire coding sequence of *PCSK9*.

In addition to testing the entire coding regions, it is important to test the structural and functional domains of proteins separately [35]. We evaluated selective pressures in different structural and functional domains by repeating the *d*_N_/*d*_S_ analyses within these domains (the domain structure of *PCSK9* is shown in Figure 1). For the C-terminal domain of *PCSK9*, the free-ratio model (*l*_1_ = −1351.35) was found to be significantly better than the one-ratio model (*l*_0_ = −1369.71, the cumulative *d*_N_/*d*_S_ = 0.386) (2Δ*l* = 36.72, *P* = 0.047, df = 24), suggesting variable selective constraint across the phylogeny for the C-terminal domain (Figure 3). We did not observe a significant difference between these two models for each of the other four domains and when the four domains were combined (data not shown).

### Non-neutral evolution of the PCSK9 C-terminal domain

Comparison of the rates of nonsynonymous and synonymous DNA changes (i.e., the ratios of *d*_N_/*d*_S_) between species can be used to assess the types of selective pressures that may have acted on a gene [36]. In the entire *PCSK9* sequence, *d*_S_ exceeded *d*_N_ in most of the branches in the primate phylogeny (*d*_N_/*d*_S_<1.0) (Figure S1), indicating that functional constraint (i.e., purifying selection) might have acted on *PCSK9* throughout primate evolution. In the branch of bonobo and gorilla, the *d*_N_/*d*_S_ ratio was = 1 (i.e., neutral evolution).

We then compared the values of *d*_N_ and *d*_S_ between species in the C-terminal domain of *PCSK9* since non-homogeneity in *d*_N_/*d*_S_ ratio was noted among the primate lineages. We found that many branches of the primate phylogeny, including internal branches, showed evidence of evolution under relaxed selective constraint or positive selection (i.e., *d*_N_/*d*_S_>1.0) (Figure 3). In the Hominoid clade, *d*_N_/*d*_S_ values was infinity (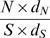: 7.1/0.0) in the lineage leading to orangutan, and 1.218 (5.1/1.1) to the common ancestors of bonobo and gorrila. The *d*_N_/*d*_S_ in the chimpanzee and human lineages approximated one [0.9890 (4.0/1.1) and 0.5657 (2.0/1.0), respectively], indicating relaxed selective constraint in these two lineages. In addition, we noted *d*_N_/*d*_S_>1 in lineages leading to colobus and rhesus macaque in the clade of Old World monkeys, as well as spider monkey, squirrel monkey, and the common ancestors of marmoset and tamarin (Figure 3). Thus, the C-terminal domain has been subject to positive selection for at least 33 million years (i.e., the primate divergence time) [37].

Then, by two-ratio likelihood tests using PAML, we tested for the presence of positive selection in the C-terminal domain of *PCSK9* in the branch of orangutan and the lineage leading to the common ancestors of bonobo and gorilla in the Hominoid clade. Log likelihood values and *d*_N_/*d*_S_ estimates from each maximum likelihood model were considered, and the likelihood ratio test results are presented in Table 2. The null hypotheses 1–3 were rejected, indicating that the *d*_N_/*d*_S_ ratio in the branch of orangutan is significantly higher than the background ratio of *d*_N_/*d*_S_ (i.e., the null hypothesis of *d*_N_/*d*_S_ ratio homogeneity among lineages was rejected). Although the alternative hypotheses of positive selection (*d*_N_/*d*_S_>1) in the branch of orangutan (null hypotheses 7 and 8 in Table 2) were not accepted at the level of 0.05, the statistical significance was marginal (*P* = 0.087 and 0.090, respectively). However, we did not observe the *d*_N_/*d*_S_ ratio in the lineage leading to bonobo and gorilla to be significantly different from the background *d*_N_/*d*_S_ ratio.

**Table 2:** Log likelihood values, parameter estimates under different models, and likelihood ratio statistics (2Δℓ**) for *d*_N_ */d*_S_ hypotheses testing.

Model	*Para* a	*ln l* b	*d*_N_ */d*_S_ (ω)			Model compared	Null hypothesis	2Δ*l*	*P* (χ^2^, df = 1)^c^
			ω_0_	ω*_h_*	ω*_g_*				
A. One ratio: ω_0_ = ω_h_ = ω_g_	27	−1369.71	0.3856	= ω_0_	= ω_0_	A vs. D	1. (ω_h_ = ω_g_) = ω_0_	6.24	0.012*
B. Two ratios: ω_0_ = ω_h_, ω_g_	28	−1366.30	0.3503	= ω_0_	Inf	A vs. B	2. ω_g_ = ω_0_	6.82	0.009*
C. Two ratios: ω_0_ = ω_g_, ω_h_	28	−1369.21	0.3686	1.0106	= ω_0_	C vs. E	3. ω_g_ = ω_0_	7.02	0.008*
D. Two ratios: ω_0_, ω_h_ = ω_g_	28	−1366.59	0.3314	2.6347	= ω_h_	A vs. C	4. ω_h_ = ω_0_	1.00	0.317
E. Three ratios: ω_0_, ω_h_, ω_g_	29	−1365.70	0.3326	1.0027	Inf	B vs. E	5. ω_h_ = ω_0_	1.20	0.273
F. Two ratios: ω_0_ = ω_h_, ω_g_ = 1	27	−1367.76	0.3502	= ω_0_	1.0000	D vs. H	6. (ω_h_ = ω_g_)≤1	1.10	0.394
G. Two ratios: ω_0_ = ω_g_, ω_h_ = 1	27	−1369.21	0.3686	1.0000	= ω_0_	B vs. F	7. ω_g_≤1	2.92	0.087†
H. Two ratios: ω_0_, ω_h_ = ω_g_ = 1	27	−1367.14	0.3324	1.0000	1.0000	E vs. I	8. ω_g_≤1	2.88	0.090†
I. Three ratios: ω_0_, ω_h_, ω_g_ = 1	28	−1367.14	0.3324	1.0140	1.0000	C vs. G	9. ω_h_≤1	0.00	1.000
J. Three ratios: ω_0_, ω_h_ = 1, ω_g_	28	−1365.70	0.3326	1.0000	Inf	E vs. J	10. ω_h_≤1	0.00	1.000

Note. – ω_h_ is *d*_N_ */d*_S_ ratio in lineage leading to the common ancestor of gorilla and bonobo; ω_g_ is *d*_N_ */d*_S_ ratio in lineage leading to orangutan; ω_0_ is the background *d*_N_ */d*_S_ ratio.

a The number of parameters estimated in the model.

b Log likelihood values.

c *P* values were obtained from the difference in the two log likelihood values of two models (2Δ*l*) with χ^2^ distribution (df = 1).

* Significant (P<0.05); ^†^ Significance is marginal (*P*<0.10).

**Figure 4:**
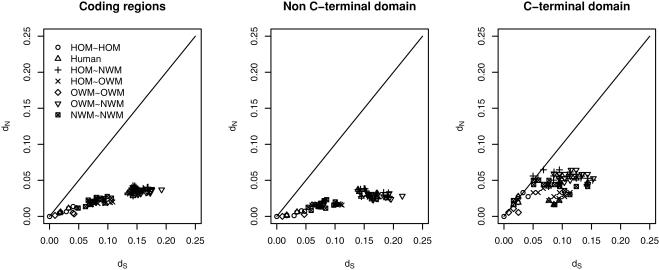
Positive selection or relaxed selective constraint of the C-terminal domain of *PCSK9*. *d*_N_ is plotted versus *d*_S_ for all pairwise combinations of primate sequences. The pairwise ratios of *d*_N_/*d*_S_ were calculated using the Nei-Gojobori method implemented in the package ‘PAML’ [72]. Pairwise combinations of Hominoids (HOM), Old World monkeys (OWM), and New World monkeys (NWM) are plotted; for example, ‘Human’ represents the points that are making comparisons between human and another primate. We plotted the entire sequence, non C-terminal domain, and C-terminal domain separately. The higher pairwise *d*_N_/*d*_S_ ratio in the C-terminal domain suggests that this domain is evolving in a non-neutral model, which maybe due to positive selection or relaxed selective constraint in some lineages. The entire sequence and non C-terminal domain of *PCSK9* showed a net signature of purifying selection.

To find out whether the C-terminal domain was under relaxed selective constraint or positive selection, we also plotted *d*_N_/*d*_S_ ratios estimated by Nei-Gojobori method for pairwise comparisons within the primates (Figure 4). In the whole gene sequence and non C-terminal domains region, *d*_N_/*d*_S_ was <1 in all pairwise comparisons, indicating a history of functional selective constraint. However, a higher *d*_N_/*d*_S_ value was noted in the C-terminal domain, including *d*_N_/*d*_S_>1 in five out of 10 pairwise comparisons within Hominoids. For example, a *d*_N_/*d*_S_ ratio of 1.122 was noted in the human vs. orangutan comparison.

### Amino acids sites under positive selection

Finally, we identified the particular codon sites that have been subject to positive selection using the site-models [38], [39] and the branch-site models [40]. Table 3 lists the log likelihood values and parameter estimates for the C-terminal domain of *PCSK9* under several site models and branch-site models. We used two likelihood ratio tests (LRTs) to test for positive selection. In the site models, the first test compared M1a (neutral) against M2a (selection) [38], [40], [41], in which 2Δ*l* is 0.68 (df = 2, *P*>0.05), and no amino acid sites were under positive selection. The second test compared M7 against M8, in which no sites were shown under positive selection (2Δ*l* = 1.66, *P*>0.05). We also used the branch-site model A to detect the codon sites under positive selection by the Bayes Emprical Bayes (BEB) approaches in the lineage of orangutan (i.e., the foreground lineage) [40]. The 2Δ*l* between the null model (neutral, *l* = −1366.56) and the alternative model (selection, *l* = −1363.55) is 6.02. The critical values at the 5% and 1% levels for the LRT are 2.71 and 5.41, respectively [42]. Thus, the test for the branch-site model A is significant (df = 2, *P*<0.01), indicating presence of codon sites under positive selection (544A, 551H, 556G, 661V, and 681S, *P_b_*>95%) in the C-terminal domain of *PCSK9*.

**Table 3:** Log likelihood values and parameter estimates under the site models and branch-site models.

Model	*Para* a	*ln l* b	Estimates of parameters	2Δ*l* c	Positively selected sites^d^
M0 (one-ratio)	1	−1369.71	ω*_0_* = 0.3856		not allowed
**Site models**					
M1a (Nearly Neutral)	2	−1366.56	*p*_0_ = 0.7704 (*p*_1_ = 0.2296); ω_0_ = 0.2110 (ω_1_ = 1)		not allowed
M2a (Positive Selection)	4	−1366.22	*p*_0_ = 0.9032; *p*_1_ = 0 (*p*_2_ = 0.0968); ω_0_ = 0.2736 (ω_1_ = 1), ω_2_ = 1.7797	(M1a vs. M2a) 0.68	none
M7 (beta)	2	−1367.06	*p* = 0.6432; *q* = 0.9806		not allowed
M8 (beta & ω)	5	−1366.23	*p*_0_ = 0.9054; *p* = 37.7260; *q* = 99.0000 (*p*_1_ = 0.0947); ω = 1.7952	(M7 vs. M8) 1.66	none
**Branch-site models**					
M1a	2	−1366.56	*p*_0_ = 0.7704 (*p*_1_ = 0.2296); ω_0_ = 0.2110 (ω_1_ = 1)		
Model A	4	−1363.55	*p*_0_ = 0; *p*_1_ = 0.7632 (*p*_2_ = 0.2368); ω_0_ = 0.1827 (ω_1_ = 1), ω_2_ = Inf	(M1a vs. Model A) 6.02**	544A, 551H, 556G, 661V, 681S

a The number of parameters estimated in the model.

b Log likelihood values.

c LRT to detect positive selection ^**^ *P*<0.01.

d Amino acid sites under positive selection based on a Bayes Empirical Bayes (BEB) probability >95%. No amino acids sites under the model M2a and M8 were shown to be under positive selection.

### Sliding window analysis

**Figure 5:**
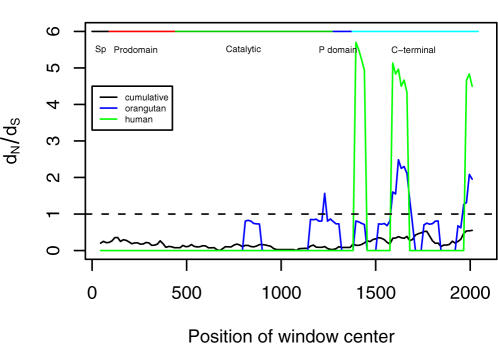
Sliding-window analysis of the cumulative *d*_N_/*d*_S_ across primates (black), the lineage leading to human (green), and orangutan (blue). The gene average *d*_S_ across primates is 0.5680, in the lineage leading to human is 0.0030, and orangutan is 0.0204.

The *d*_N_/*d*_S_ profiles in the sliding window analysis across *PCSK9* sequence are shown in Figure 5. As expected, the cumulated *d*_N_/*d*_S_ ratio in primates in the sliding window analysis appear quite stochastic and bears weak correlation to the domain structure, although the *d*_N_/*d*_S_ is slightly higher in the C-terminal domain. However, in the lineages leading to humans and orangutan, we observed that nonsynonymous substitutions were significantly more concentrated within C-terminal domain of *PCSK9* (i.e., three *d*_N_/*d*_S_ peaks). The *d*_N_/*d*_S_ peaks were consistent between the lineage leading to humans and to orangutan.

## Discussion

The main finding of our study is that there is evidence for functional constraint (i.e., purifying selection) in the coding sequences of *PCSK9* through primate evolution. We noted that a functional domain of *PCSK9* (i.e., C-terminal domain) was less conserved at the amino acid level than other gene regions, and likelihood ratio tests (LRTs) revealed evidence of positive selection in the lineage leading to orangutan of the Hominoid clade on this domain. Furthermore, we identified the particular codon sites that have been subject to positive selection in this lineage. We discuss the implications of these comparative sequence data for understanding the evolutionary history of primate *PCSK9*, hypotheses concerning their role in primate phenotypic evolution, and insights into *PCSK9*-associated human diseases.

### Evolutionary history of Leucine (L) repeats in the signal peptide (SP) domain and premature stop codon in the C-terminal domain

Comparative sequence analysis revealed a dynamic evolutionary history of leucine (Leu, L) repeats in the signal peptide domain. The number of Leu repeats varied from L9 to L6 among different clades (Figure 2). An additional in-frame insertion (CTG) leading to a L9→L10 polymorphism in African-Americans and Caucasians [19] is associated with hypocholesterolemia. We speculate that the number of Leu repeat may influence levels of the *PCSK9* protein and thereby levels of LDL cholesterol, although this needs confirmation.

In the C-terminal domain of *PCSK9*, a premature stop codon was seen in the New World monkeys – tamarin and dusky titi (Figure 2) – but not in the Hominoids. As mentioned before, monkeys have significantly lower LDL cholesterol levels than Hominoids [22]. It is unknown whether the loss-of-function mutation in the C-terminal domain is a random phenomenon or a common feature that influences cholesterol metabolism. A premature stop codon mutation in human *PCSK9* (C679X) is considered to be under positive selection, and it is speculated that loss of *PCSK9* function interferes with the life cycle of the malaria parasite through cholesterol restriction [43], [44]. The “less-is-more” hypothesis of Olson [45] posits that loss of gene function during hominoid evolution may in some cases have conferred a fitness benefit and led to adaptive evolution that may help explain differences among primates [46].

### Amino acid substitution patterns

The *d*_N_/*d*_S_ ratio for the coding regions of *PCSK9* across the primate species was <1 (cumulative *d*_N_/*d*_S_ = 0.186). There were no lineages with *d*_N_/*d*_S_>1, and *d*_N_/*d*_S_ ratios did not vary among branches (*P* = 0.480, Figure S1). This is not unexpected, as averaging *d*_N_/*d*_S_ across all sites is not a powerful test of adaptive evolution [39]. However, the nonsynonymous substitution rate in the C-terminal domain was significantly higher than in other domains. The cumulative *d*_N_/*d*_S_ = 0.386 is higher than that in the entire coding region (*d*_N_/*d*_S_ = 0.186), suggesting that different selection pressures have acted on amino acid changes across different functional regions of this gene.

The hypothesis of *d*_N_/*d*_S_ homogeneity among branches was rejected for the C-terminal domain (*P* = 0.047, Figure 3), which could reflect either relaxed selective constraint or positive selection for amino acid substitution along one or more lineages. We were particularly interested in two lineages in the Hominoid clade leading to orangutan (*d*_N_/*d*_S_ = infinity), as well as the common ancestor of bonobo and gorilla (*d*_N_/*d*_S_ = 1.213), and tested whether the *d*_N_/*d*_S_ ratio was significantly >1 on these two branches. Likelihood ratio tests (LRTs) from a two-ratio model revealed that positive selection (*d*_N_/*d*_S_>1) had acted on the orangutan lineage although the statistical evidence was marginal (*P* = 0.087 and *P* = 0.090) (Table 2). However, the pattern of *d*_N_/*d*_S_ heterogeneity across lineages is consistent with a relaxed selective constraint. The recently developed branch-site model A is powered to detect the particular amino acid sites that have been subject to positive selection in a given lineage (i.e., a foreground branch) [40], [47], and at least five positively selected amino acids of the C-terminal domain existed in the lineage leading to orangutan (Table 3). No amino acids under positive selection were detected using site models (Table 3). It should be noted that the power of the LRTs is dependent on the number of coding sequences [48]. We sampled 14 primate species in Hominoid, Old World monkey, and New World monkey clades in our phylogenetic analyses (Table 1). A greater number of the species might have permitted more robust inferences of positive selection on the C-terminal domain of *PCSK9*.

The sliding-window analysis ratio further characterized the non-random nonsynonymous substitution along *PCSK9* and *d*_N_/*d*_S_ peaks were obvious in the C-terminal domain (Figure 5). Although the *d*_N_/*d*_S_ ratio in the lineage of humans is <1 (*d*_N_/*d*_S_ = 0.566), three striking peaks of *d*_N_/*d*_S_ (> 4) in human lineage were noted in the C-terminal domain. However, these peaks could be partly explained on the basis that human *PCSK9* shows very little synonymous divergence (*d*_S_ = 0.003).

In the present study, we calculated the *d*_N_/*d*_S_ ratios across the phylogenetic tree using the ‘gene’ tree instead of the ‘species’ tree. We also performed analyses using the ‘species’ tree [49], in which bonobo is most closely related to chimpanzee and gorilla is sister to the human/chimpanzee clade. Although the log likelihood values (*l*_0_ for one-ratio model and *l*_1_ for free-ratio model) under the ‘species’ tree were different from that under the ‘gene’ tree, we did not find a significant difference in the *d*_N_/*d*_S_ ratio between the ‘species’ tree and ‘gene’ tree for the coding regions and C-terminal domain. Although we did not observe *d*_N_/*d*_S_ ratio >1 in the lineage leading to bonobo or gorilla in the C-terminal domain, we did note *d*_N_/*d*_S_ = infinity in the lineage leading to orangutan. In addition, we used the branch-site A model to test for positive selection in the lineage leading to orangutan based on the species tree. The 2Δ*l* between the null model (neutral, *l* = −1408.2) and the alternative model (selection, *l* = −1406.7) is 3.0 (df = 2, *P*<0.05) (critical value is 2.71 at 5% significance level [42]). We detected three codon sites under positive selection (544A, 551H, and 681S, *P_b_*>95%) in the C-terminal domain of *PCSK9* (using BEB analysis). Zhang et al. [47] suggested a critical value of 3.84 (for *P*<0.05), however, such a threshold may be too conservative for a sequence length of 200 codons [47].

### Structural and functional implication of PCSK9

The correct folding of the C-terminal domain is crucial for *PCSK9* function but catalytic activity is not required for *PCSK9* to bind and degrade LDL-R in cultured human hepatoma cells [50]. The C-terminal domain of a proprotein convertase contains unique sequences regulating their cellular localization and trafficking [51]. For example, *PCSK9* exhibits a Cys-His-rich domain that is required for cell surface binding in an LDL-R-dependent fashion [6] and plays a role in the regulation of auto-processing of *PCSK9*. The structural characteristic of C-terminal domain may determine the colocalization of *PCSK9* with LDLR at the cell surface [52] or lead to other novel functional properties. Hence, positive selection operating on the C-terminal domain was most likely directed at creating novel biochemical properties.

Species-specific differences in *PCSK9* expression patterns have been noted in brain and liver among humans, chimpanzee, and orangutan [53]. *PCSK9* is transiently expressed during embryonic development in telencephalon and cerebellum where *LDLR* expression is not prominent [6], [54]. Specific knockdown of *Pcsk9* mRNA led to embryonic death at 4 days after fertilization in zebrafish [54], and complete knockout of *Pcsk9* in mouse led to a ∼50% reduction in circulating levels of LDL cholesterol, but did not result in a lethal phenotype [55]. Over-expression of *PCSK9* induces apoptosis in neural development [6], [56], which results in a higher percentage of differentiated neurons and promotes cortical neurogenesis. These results indicate a novel function of *PCSK9* in central nervous system development, distinct from that in cholesterogenic organs such as liver [54]. One could hypothesize that relaxed selective constraint or positive selection has operated on the C-terminal domain of *PCSK9* due to the key role of *PCSK9* in early brain development.

### Implications for human diseases

There is increasing interest in identifying gene loci affected by natural selection since they are medically important [57]–[63]. Loss-of-function or gain-of-function mutations in *PCSK9* have been reported to be associated with significant alterations in plasma levels of LDL cholesterol (Figure 1). Both evolutionary conservation indicating negative purifying selection and accelerated evolution driven by positive selection signify functionally significant regions of the genome [64]. To assess the potential severity of human *PCSK9* mutations, we assessed the levels of conservation or divergence of non-synonymous mutations listed in Figure 1 by aligning the amino acids among 14 species (Figure 2). We expected that the amino acids known to be important for *PCSK9* function (i.e., residues at which disease-causing mutations occur) would be highly conserved. All gain-of-function mutations in *PCSK9* leading to hypercholesterolemia in humans are 100% conserved at the amino acid level across all the primates we sampled. In case of loss-of-function mutations leading to hypocholesterolemia, all but two (E57K and Q554E) are 100% conserved across the primates. Mutations leading to both hypercholesterolemia and hypocholesterolemia appear to be less conserved, since four out of seven such mutations are not all conserved, including A53V, I474V, Q554E, and E670G. We noted a striking pattern of I474V variation (SNP rs562556) across the primates. The ancestral state of the 474^th^ amino acid (M or V) in New World monkeys is not clear given the lack of an outgroup. The ‘V’ allele diverged to ‘I’ or ‘V’ in the Hominoid clade, suggesting a dynamic evolutionary history of the 474^th^ amino acid. The human mutation I to V replicates the ancestral state, and the recurrence of this ancestral state has functional consequences [65].

To survey polymorphisms within human populations, we analyzed *PCSK9* SNPs using resequenced data from 24 African-Americans and 23 European-Americans (i.e., 47 individuals) in SeattleSNPs database (pga.gs.washington.edu/). A total of 229 polymorphic sites in African-Americans and 125 polymorphic sites in European-Americans were found in the human panel, eight of which resulted in amino acid changes. In addition, an in-frame insertion/deletion (CTG) in the signal-peptide domain was noted in both populations. Six of the eight non-synonymous sites are located in the putative domain and C-terminal domain (some investigators combine these two domains as ‘C-terminal’ domain), corresponding to the regions that have been predicted to be under positive selection. None of the non-synonymous sites was found in the catalytic domain. We used SIFT [66] and PolyPhen [67] to predict the effect of the amino acid changes (Table S2). In case of amino acid 474, the nonsynonymous substitution was predicted to be damaging (i.e., cause functional alteration), but the derived allele frequency of 0.79 in African-Americans and 0.87 in European-Americans suggests that positive selection acted to increase the frequency of this polymorphism. In humans, a signature of recent positive selection was noted on this common variation using long-range haplotype (LRH) test; that is, positive selection had acted on the derived allele ‘I’ in African-Americans and the ancestral allele ‘V’ in European-Americans (Ding and Kullo, manuscript in revision). In addition, a signature of positive selection on the derived allele of E670G (rs505151), which resides in the C-terminal domain, was also noted in African-Americans. We speculate that non-conserved mutations across the primates might be the substrate for non-neutral evolution and responsible for the phenotypic variation in the general population.

In conclusion, phylogenetic analysis of the cholesterol metabolism gene *PCSK9* across a range of primates reveals lineage-specific patterns of variation. Although the gain-of-function mutations at *PCSK9* reflect strong functional constraint and a history of purifying selection, a signature of relaxed selective constraint or positive selection was noted in the C-terminal domain of *PCSK9*. It is possible that different modes of selection have operated on different functional domains of *PCSK9*.

## Materials and Methods

### Primate Genomic DNA Sources

The comparative sequences of the *PCSK9* coding regions were obtained in 14 species from three sources. First, the human (accession no.: NM_174936) and chimpanzee (XM_427085) mRNA sequences of *PCSK9* were downloaded from NCBI (www.ncbi.nlm.nih.gov). Next, we acquired the BAC (bacterial artificial chromosome) clone sequence including *PCSK9* from Programs for Genomic Application (PGA) at Berkeley (pga.lbl.gov/seq), including colobus (AC188217), dusky titi (AC188268), squirrel monkey (AC188233), and marmoset (AC188221). Coding regions of *PCSK9* for these species were extracted by aligning the human mRNA sequence to the BAC sequence using the ‘sim4’ program [68]. Finally, DNA samples for a primate panel, including rhesus macaque, pigtailed macaque, bonobo, gorilla, chimpanzee, orangutan, tamarin, spider monkey, woolly monkey, and lemur, were obtained from Coriell Cell Repositories (Camden, NJ). The species name and scientific name for each species are listed in Table 1.

### Sequencing of PCSK9 exons from Primate Genomic DNA

In the primate panel, *PCSK9* was amplified and sequenced exon by exon from genomic DNA with high fidelity polymerase chain reaction (PCR). Primers and PCR conditions are listed in Table S1. PCR products were sequenced directly in both forward and reverse directions. Exon reads were assembled together to create virtual transcript for each primate using the Sequencher® program (version 4.5, www.genecodes.com) and visually checked for accuracy. The lemur *PCSK9* sequence could not be obtained due to difficulty in PCR amplification. Sequences of coding regions for eight species in this primate panel were obtained. A total of 2072 bp of *PCSK9* coding sequence (the length is based on the human sequence and excludes the stop codon) in 14 species was compiled. All sequences have been submitted to the GenBank database under the accession nos. EF692496–EF692509 (Table 1).

### Detecting lineage-specific episodes of positive selection

Sequences were aligned using ClustalW [69], followed by manual inspection and analysis. We used the ‘HYPHY’ package to estimate the topology of phylogenetic tree using the maximum likelihood method [34]. Since the gene tree was different from the species tree, analyses were done based on gene tree as well as the species tree.

We used the maximum likelihood method based on codon-substitution model by Yang [28], [31], [70] to test whether there was a significant difference in *d*_N_/*d*_S_ ratio (i.e., ω) among lineages and whether *d*_N_/*d*_S_ was significantly >1 (i.e., positive selection) in a given lineage. The ‘one-ratio’ model assumes the same ratio for all branches in the phylogeny. The most general model – ‘free-ratio’ model – assumes an independent *d*_N_/*d*_S_ ratio for each branch in the phylogeny. If there is a phylogenetic tree of many species, this model involves as many *d*_N_/*d*_S_ parameters as the number of branches in the tree. The models used in the phylogenetic analysis can be compared using the likelihood-ratio test to examine interesting hypotheses [31]. The null hypothesis is the ‘one-ratio’ model, and can be used to test whether there is a differential *d*_N_/*d*_S_ ratio among lineages. Positive selection or relaxed selective constraint in some lineages could contribute to the heterogeneity in the *d*_N_/*d*_S_ ratio.

### Detecting amino acid sites under positive selection

The above methods for lineage-specific selection assume that all amino acid sites have the same *d*_N_/*d*_S_ ratio, i.e., averages the *d*_N_/*d*_S_ ratio across all sites. Since many amino sites might be under strong purifying selection due to functional constraint (*d*_N_/*d*_S_≈zero) and positive selection often operates episodically on a few amino acid sites [47], it seems likely that this is a more conservative test and amino acid sites under positive selection cannot be detected.

Several methods have been developed to address this problem, such as the site models which allow *d*_N_/*d*_S_ to vary among codons [38], [71]. In the present study, we also used an improved branch-site likelihood method to detect positive selection at the amino acid sites [28], [40], [47]. This branch-site model [28], [40], [47] assumed that the branches on the phylogeny are divided *a priori* into foreground (i.e, may have experienced positive selection) and background lineages. We used the likelihood-ratio test 2 (i.e., the branch-site test of positive selection) constructed from this branch-site model [47]. The null hypothesis of this LRT is the branch-site model A list above but with ω_2_ fixed = 1, which can be used to directly test for positive selection on the foreground lineages [47]. The Bayes empirical Bayes (BEB) approach was used to calculate the posterior probabilities that a codon belongs to the site class of positive selection on the foreground lineages [40]. The test should be compared with the 50∶50 mixture of point mass 0 and 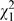 (with critical values to be 2.71 and 5.41, at the 5% and 1% significance levels, respectively) [42]. Zhang et al [47] also suggested the use of 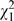 distribution for assessing the significance of the test (3.84 and 5.99 at the 5% and 1% significance levels, respectively). This LRT test seemed conservative overall, but exhibited better power in detecting positive selection than the branch-based test [47].

We used the ‘CODEML’ program in PAML version 3.15 [72] to calculate the *d*_N_/*d*_S_ ratio and perform the maximum likelihood phylogenetic analysis. To calculate the *d*_N_/*d*_S_ ratio at lineages (defined as all branches in the phylogeny, both terminal species nodes and internodes), sequences associated with species-specific premature stop codons were removed.

### Sliding-window analysis of d_N_/d_S_

Sliding-window analysis of *d*_N_ */d*_S_ was performed with a window size of 90 bp (30 codons) and a sliding increment of 15 bp (5 codons). We used the approach by Choi and Lahn [73] to calculate the *d*_N_ */d*_S_ of each window as the ratio between window-specific *d*_N_ and gene-average *d*_S_, since noise in window-specific *d*_S_ can sometimes hamper the analysis. In addition, the use of gene-average instead of window-specific *d*_S_ should not introduce any systematic bias [73].

## Supporting Information

Figure S1 Phylogeny of coding regions of *PCSK9*. *PCSK9* was resequenced from a panel of primates including Hominoids, Old World monkeys, and New World monkeys. Branch lengths were estimated by maximum likelihood under the free-ratio model, which assumes an independent *d*_N_/*d*_S_ ratio for each branch.
(0.01 MB EPS)

Table S1 Polymerase chain reaction (PCR) primers and conditions for *PCSK9* exon analyses
(0.10 MB DOC)

Table S2 SIFT and Polyphen prediction of amino acid polymorphisms
(0.04 MB DOC)
