# Supplementary material for: Evidence for Positive Selection in the C-terminal Domain of the Cholesterol Metabolism Gene PCSK9 Based on Phylogenetic Analysis in 14 Primate Species
Source: PLoS One. 2007 Oct 31;2(10):e1098. doi: 10.1371/journal.pone.0001098 (PMC2034530; doi:10.1371/journal.pone.0001098)
Supplement: Table S1 — Polymerase chain reaction (PCR) primers and conditions for PCSK9 exon analyses (0.10 MB DOC) [file pone.0001098.s002.doc]

**Table S1.** Polymerase chain reaction (PCR) primers and conditions for *PCSK9* exon analyses

| **Primer set** | **Primer** | **5' primer sequence 3'** | **Species** |
| --- | --- | --- | --- |
| Exon1_A | Exon1_F | AACTTCAGCTCCTGCACAGT | G, SM, PM, WM, B, RM, T, O |
|  | Exon1_R | AACTGAGGCCCGAGAGGAAA |  |
|  |  |  |  |
| Exon2_A | Exon2_F | TCATCATGTTCCTCCTTGCA | G, SM, PM, B, RM, T, O |
|  | Exon2_R | CCCTATCAGGAAGTGCCATT |  |
| Exon2_B | Exon2_F2 | TGAGATAAAGTACACATAGG | WM |
|  | Exon2_R | CCCTATCAGGAAGTGCCATT |  |
|  |  |  |  |
| Exon3_A | Exon3_F | AACTCAGGCTCCTAGTCTGT | G, PM, B, RM, O |
|  | Exon3_R | AGCAAATGGATTCAGCTCAG |  |
| Exon3_B | Exon3_F2 | TGCTGTCCAGATGGCTTAAG | SM |
|  | Exon3_R | AGCAAATGGATTCAGCTCAG |  |
| Exon3_C | Exon3_F2 | TGCTGTCCAGATGGCTTAAG | WM |
|  | Exon3_R2 | AGAAGTGGAAACCACTGGCA |  |
| Exon3_D | Exon3_F3 | ACAGGTGTGATCAGGTGAGG | T |
|  | Exon3_R2 | AGAAGTGGAAACCACTGGCA |  |
|  |  |  |  |
| Exon4_A | Exon4_F | ATGCTCATTCCCTCCTCTCC | G, PM, B, RM, O |
|  | Exon4_R | ACCTCCAGGATGGGGATAT |  |
| Exon4_B | Exon4_F | ATGCTCATTCCCTCCTCTCC | SM, T |
|  | Exon4_R2 | AGTACAGCTGCAACACTCTT |  |
| Exon4_C | Exon4_F2 | AGGATCTAGCGTGCGAGGAT | WM |
|  | Exon4_R2 | AGTACAGCTGCAACACTCTT |  |
|  |  |  |  |
| Exon5_A | Exon5_F | TCTCATGTGGTCCTTGTGTT | G, SM, PM, B, RM, O |
|  | Exon5_R | TCCAGATGGAGAGAGACCA |  |
| Exon5_B | Exon5_F2 | TTCATCCATCCAGCCACCTG | WM, T |
|  | Exon5_R2 | TTAGGAGACATTAGCTCTCC |  |
|  |  |  |  |
| Exon6_A | Exon6_F_O | TCCTCTGTGCCTGTAAGGGA | G, B |
|  | Exon6_R_O | AGCAGCCCCAGCACCTA |  |
| Exon6_B | Exon6_F2 | TGACCAAACATCAGGCACA | O |
|  | Rxon6_R2 | TCCAGGAACGTGCCACAAGAA |  |
| Exon6_C | Exon6_F3 | AACCTGCCTTACTCAGTCTC | SM, PM, WM, RM |
|  | Exon6_R3 | GTCACTGCTGTTCAGACTCT |  |
| Exon6_D | Exon6_F4 | TAACCATCACTCTGTGCCTG | T |
|  | Exon6_R4 | TTCCAAAGCCAGAAGGGTT |  |
|  |  |  |  |
| Exon7_A | Exon7_F | CCTCTCTTGGGCTCCTTTCT | G, PM, B, RM, O |
|  | Exon7_R | AGGCAGTGGGTGGTGACTTA |  |
| Exon7_B | Exon7_F | CCTCTCTTGGGCTCCTTTCT | WM, T |
|  | Exon7_R2 | TGTTAGCATCACGGTGGCCA |  |
| Exon7_C | Exon7_F3 | TGTATAGCAGTTGTTCAGCC | SM |
|  | Exon7_R3 | ATCAGGCCTGTCTCATCTC |  |
|  |  |  |  |
| Exon8_A | Exon8_F | ACCATCTTTCACCATTCACC | G, SM, PM, B, RM, O |
|  | Exon8_R | TGTCAAGGTCACACAGACCT |  |
| Exon8_B | Exon8_F | ACCATCTTTCACCATTCACC | WM, T |
|  | Exon8_R2 | ATCCTCCTTACACAGACAAG |  |
|  |  |  |  |
| Exon9_A | Exon9_F | TTTAAGCCCTCCTCTCTCCT | G, B, O |
|  | Exon9_R | AAGGAGGGGTACAGTCAC |  |
| Exon9_B | Exon9_F2 | CTCTCTCCTACCATGAACTA | PM, RM |
|  | Exon9_R2 | AAGAGCTGGAGTGTCGAGGA |  |
| Exon9_C | Exon9_F3_SM | TTGTCTGTGTAAGGAGGATG | SM, WM |
|  | Exon9_R3_SM | ATCCAGCATTGTCCTGTAGCC |  |
| Exon9_D | Exon9_F3 | TAAGGAGGATGACACCACCT | T |
|  | Exon9_R | AAGGAGGGGTACAGTCAC |  |
|  |  |  |  |
| Exon10_A | Exon10_F | AGCAGATTCCCATTTCCGTC | G, WM, B, O |
|  | Exon10_R | TGCATAAGGAGAAAGAGACC |  |
| Exon10_B | Exon10_F2 | TGCTTGAGTTGATCCTGTCT | PM, RM |
|  | Exon10_R2 | TCTCATGGATCACACTCATG |  |
| Exon10_C | Exon10_F2 | TGCTTGAGTTGATCCTGTCT | SM |
|  | Exon10_R3 | GAAGCTGAGATGAGGAGAAG |  |
| Exon10_D | Exon10_F3 | ACCACTCTGTGTTTGGAAGA | T |
|  | Exon10_R3 | GAAGCTGAGATGAGGAGAAG |  |
|  |  |  |  |
| Exon11_A | Exon11_F | AGACGGAGCATCCCAGCATT | G, PM, B, RM, O |
|  | Exon11_R | TATGGTGGTGGCACAAACTG |  |
| Exon11_B | Exon11_F2 | TAGCTCTTGCCTCAGACCTT | SM, WM |
|  | Exon11_R | TATGGTGGTGGCACAAACTG |  |
| Exon11_C | Exon11_F2 | TAGCTCTTGCCTCAGACCTT | T |
|  | Exon11_R2 | ACACACTGTCCACACGATGG |  |
|  |  |  |  |
| Exon12_A | Exon12_F | TGCTTTCTTTTCCTCGGGCT | G, WM, B, O |
|  | Exon12_R | AGGGACAAGTCGGAACCATT |  |
| Exon12_B | Exon12_F2 | TTGGCCTCACAGCGGGATGTT | PM, RM, T |
|  | Exon12_R2 | CTCCAGGAAGGTGGAAGCAT |  |
| Exon12_C | Exon12_F3 | ATGAAGCAGGAGCTCACGTGT | SM |
|  | Exon12_R2 | CTCCAGGAAGGTGGAAGCAT |  |

PCR primers were originally designed in 100% conserved regions between human, chimpanzee, and rhesus macaque since the genome sequence for these species are available (genome.ucsc.edu). Following initial PCR amplification and DNA sequencing, species-specific primers were designed.

**Species abbreviation**: G, gorilla; SM, spider monkey; PM, pigtailed macaque, WM, woolly monkey; B, bonobo; RM, rhesus macaque; T, tamarin; O, orangutan

**Notes. −**Each 25 *µ*l PCR reaction mixture contained 50 ng DNA, 200 *µ*M of each dNTP, 1 *µ*M of each primer, 1.5 mM of MgCl_2_, 50 mM KCl, 10 mM Tris-HCl, 1 U of AmpliTaq Gold (Applied Biosystems) and 5% DMSO. PCR cycle conditions were performed on a 9800 Fast Thermo Cycler (Applied Biosystems).

**PCR conditions were as follows:**

Step 1: Initial denature, 95ºC for 10 min

Step 2: Denature, 95ºC for 30 sec

Step 3: Annealing, touchdown protocol, -2ºC per cycle for 30 sec

Step 4: Extension, 72ºC 45 sec

Repeat steps 2-4, for a total of 6 cycles

Step 5: Denature, 95ºC for 30 sec

Step 6: Annealing, at 55ºC for 30 sec

Step 7: Extension, at 72ºC for 30 sec

Step 8: Final extension, 70ºC for 10 min
