# Supplementary material for: Evidence for Positive Selection in the C-terminal Domain of the Cholesterol Metabolism Gene PCSK9 Based on Phylogenetic Analysis in 14 Primate Species
Source: PLoS One. 2007 Oct 31;2(10):e1098. doi: 10.1371/journal.pone.0001098 (PMC2034530; doi:10.1371/journal.pone.0001098)
Supplement: Table S2 — SIFT and Polyphen prediction of amino acid polymorphisms (0.04 MB DOC) [file pone.0001098.s003.doc]

Table S2. SIFT and PolyPhen prediction of the effect of amino acid polymorphisms

| Position | AA | | Freq. of Derived Allele^*^ | Prediction | | |
| --- | --- | --- | --- | --- | --- | --- |
|  | Ancestral | Derived |  | PolyPhen | SIFT | Location |
| 46 | R | L | 0.00, 0.02 | possibly damaging | Tolerated | SP domain |
| 53 | A | V | 0.02, 0.09 | benign | Tolerated | prodomain |
| 425 | N | S | 0.02, 0.00 | benign | Tolerated | P domain |
| 443 | A | T | 0.06, 0.00 | benign | Tolerated | P domain |
| 474 | V | I | 0.79, 0.87 | benign | Damaging | P domain |
| 553 | H | R | 0.02, 0.00 | probably damaging | Affect function | C terminal domain |
| 619 | Q | P | 0.02, 0.00 | benign | Tolerated | C terminal domain |
| 670 | E | G | 0.38, 0.02 | possibly damaging | Tolerated | C terminal domain |

^*^ frequency of derived allele in African-Americans, and European-Americans

SP, signal peptide; P domain, putative P domain ;
